# Analysis of Snail1 function and regulation by Twist1 in palatal fusion

**DOI:** 10.3389/fphys.2013.00012

**Published:** 2013-02-19

**Authors:** Wenli Yu, Yanping Zhang, L. Bruno Ruest, Kathy K. H. Svoboda

**Affiliations:** Department of Biomedical Sciences, Center for Craniofacial Research and Diagnosis, Texas A&M University, Baylor College of DentistryDallas, TX, USA

**Keywords:** palatal fusion, Snail1, Twist1, E-cadherin, Tgfβ3, E-proteins, epithelial-mesenchymal transition

## Abstract

Palatal fusion is a tightly controlled process which comprises multiple cellular events, including cell movement and differentiation. Midline epithelial seam (MES) degradation is essential to palatal fusion. In this study, we analyzed the function of Snail1 during the degradation of the MES. We also analyzed the mechanism regulating the expression of the *Snail1* gene in palatal shelves. Palatal explants treated with Snail1 siRNA did not degrade the MES and *E-cadherin* was not repressed leading to failure of palatal fusion. Transforming growth factor beta 3 (Tgfβ3) regulated *Snail1* mRNA, as *Snail1* expression decreased in response to *Tgf*β*3* neutralizing antibody and a PI-3 kinase (*PI3K*) inhibitor. Twist1, in collaboration with E2A factors, regulated the expression of *Snail1*. Twist1/E47 dimers bond to the *Snail1* promoter to activate expression. Without E47, Twist1 repressed *Snail1* expression. These results support the hypothesis that Tgfβ3 may signal through Twist1 and then Snail1 to downregulate *E-cadherin* expression during palatal fusion.

## Introduction

Secondary palatal fusion is a tightly controlled process that has been described in many reviews and research papers (Ferguson, 1988; Jugessur and Murray, 2005; Gritli-Linde, 2007; Nawshad, 2008; Yu et al., 2009). Briefly, the two palatal shelves initiate as outgrowth from the inner wall of the maxillary prominences as early as embryonic day (E) 12 in mice. They first grow lateral to the tongue and later become reoriented rostral to the tongue. At E14, the shelves contact and adhere at the midline, where the epithelium covering the tip of the palatal shelves forms a seam termed the midline epithelial seam (MES). Later, the seam breaks down to achieve mesenchymal confluence. The mechanisms for this midline MES degradation are not clear and great interest has been raised on this developmental event since failure of this process results in cleft palate.

Morphological analyses have demonstrated that, before fusion, medial edge epithelial (MEE) cells within the epithelial seam bulge and form filopodia-like structures (Taya et al., 1999; Ding et al., 2004; Fujiwara et al., 2008). Filopodia are actin-based structures associated with mesenchymal cell migration or interaction with the extracellular matrix (ECM) or other cells (Gupton and Gertler, 2007; Mattila and Lappalainen, 2008). Filopodia in MEE cells help them to correctly align and adhere to their target partner and close the gap between them, a process known as “adhesion zippering” and are used in cell guidance and migration (Taya et al., 1999; Bridgman et al., 2001; Millard and Martin, 2008). In knockout mice with disrupted Transforming growth factor beta 3 (*Tgf*β*3*) or Platelet-derived growth factor C (*Pdgfc*) signaling, the filopodia on the apical surface of MEE cells are either reduced or completely lost (Taya et al., 1999; Ding et al., 2004). The extension of the filopodia-like structures on the MEE cell's surface before or during fusion indicates that cell migration contributes to the palatal fusion (Martin-Blanco and Knust, 2001). Thus, it has been proposed that the epithelial cells migrate to the oral and nasal surface (Cuervo and Covarrubias, 2004). In addition, anterior-posterior migration of the cells has also been proposed (Jin and Ding, 2006). Epithelial cell migration often involve their transformation into mesenchymal cells (Yang et al., 2006).

Studies with cell tracking dyes demonstrated that during fusion, the MEE are viable and are separating from the seam as mesenchyme cells (Fitchett and Hay, 1989). Some of the labeled epithelial cells were found in the mesenchyme after fusion (Griffith and Hay, 1992), indicating that epithelial-mesenchymal transition (EMT) occurred. *Tgf*β*3*, by activating PI-3 kinase (PI3K) signaling, has been considered a “master gene” in initiating EMT and regulating MEE cell fate (Hay, 1989; Kang and Svoboda, 2002; Nawshad et al., 2004, 2005). Cultured MEE cells treated with Tgfβ3 undergo EMT, migration and apoptosis in that chronological sequence (Ahmed et al., 2007). However, EMT during palate fusion remains controversial (Vaziri Sani et al., 2005; Dudas et al., 2006; Jin and Ding, 2006; Xu et al., 2006). While some did not find evidence of EMT during secondary palate fusion (Dudas et al., 2006), a MEE cell fate mapping study by Jin and Ding (2006) revealed its presence. However, most of the cells that underwent the transformation eventually died after migrating away from the seam and only a few persisted, probably explaining the contradictory results. The sequential events observed in Jin and Ding study are in agreement with the *in vitro* study by Ahmed and colleagues (2007). The transformation may be necessary to maintain the fusion suture patency (Jin and Ding, 2006).

Previously, we demonstrated that the basic-helix-loop-helix (bHLH) transcription factor, Twist1 protein is expressed intensively in the MEE cells right before fusion while also expressed in the mesenchyme (Yu et al., 2008), which was confirmed by another group (Kitase et al., 2011). Down regulation of *Twist1* using siRNA in palatal organ culture resulted in blocked fusion (Yu et al., 2008). In addition, Twist1 was increased in Tgfβ3 treated chicken palatal shelves and downregulated when mouse palates were treated with neutralizing antibodies against Tgfβ3 (Yu et al., 2008).

*Twist1* has been implicated as an EMT regulator. The *Twist1* role in tumor progression notably sustains and enhances this theory (Yang et al., 2004). However, *Twist1*-null heterozygous mice (*Twist1*^+/−^) exhibited phenotypes similar to the dominantly inherited Saethre–Chotzen syndrome in the human population (Bourgeois et al., 1998) with a low penetrance of cleft palate (Stoler et al., 2009), indicating that there are other factors compensating for its function *in vivo*.

Like *Twist1*, the *Snail1* gene is well-documented for its evolutionarily conserved roles in mesoderm development and has been implicated in several cellular events such as EMT, cell migration, and survival (Cano et al., 2000; Barrallo-Gimeno and Nieto, 2005). *Snail* genes encode DNA binding zinc-finger proteins that act as transcriptional repressors (Carver et al., 2001). *Snail1* is expressed in the palatal and dental mesenchyme adjacent to the epithelium (Rice et al., 2005). In addition, *Snail1* mRNA was also found in a small subpopulation of the MEE cells after the seam had formed (Martinez-Alvarez et al., 2004). Transgenic mice have provided insights into function of this gene family in palatogenesis. Conditional deletion of the *Snail1* gene in neural crest cells did not cause obvious deformities in the craniofacial region unless the mouse was bred with a *Snail2*^−/−^ mouse (Murray et al., 2007), suggesting that Snail2 may compensate for the loss of Snail1 function. However, the role of Snail1 in epithelial cells has not been fully investigated.

A hierarchical relation between them was proposed based on the evidence that *Twist* was required for *Snail* mRNA expression and *Snail* was required for the maintenance of *Twist* expression during Drosophila mesoderm formation (Brouzes et al., 2004). Twist1 dimerizes with *E2A*-encoded proteins E12 and E47, for successful EMT (Perez-Moreno et al., 2001). The target sequence of these bHLH proteins is the E-box. Interestingly, Snail1 binds to the same consensus sequence on the *E-cadherin* promoter and acts as a repressor in EMT (Batlle et al., 2000; Cano et al., 2000; Oram and Gridley, 2005). Snail1 may compete directly with bHLH proteins for the same binding sequences (Oram and Gridley, 2005). However, Snail1 also cooperates with Twist1 to inhibit the expression of *p21-cip1* induced by *E2A*-gene products in osteoblast-like cell differentiation (Takahashi et al., 2004). Collectively, the functional networks between Snail1, Twist1, and E2A proteins in cell differentiation and movement remain to be elucidated.

In this study we used a variety of approaches to determine if Snail1 has a function in EMT and palatal fusion. In the presence of *Snail1* siRNA, E-cadherin expressing MEE remained at the palatal fusion site, suggesting Snail1 was responsible for *E-cadherin* down regulation during MES degradation. *Snail1* expression was decreased in response to the Tgfβ3 neutralizing antibody and PI3K inhibitor during palatal fusion. In addition, we used transfected cell cultures with luciferase detection to test if Twist1 cooperates with E proteins to regulate the *Snail1* promoter activity. Our results support the hypothesis that Twist1 may regulate MES degradation during palatal fusion partially through *Snail1* regulation.

## Materials and methods

### Animal manipulation, palatal organ culture, and cell culture

The protocol for the use of animals was approved by the Institutional Animal Care and Use Committee at Baylor College of Dentistry, and the animals were euthanized following NIH guidelines. Timed-pregnant CD1 mice (Harlan Sprague-Dawley, Inc.) and fertile chicken eggs (Texas A&M Poultry Science Department) were used in these studies. Mouse embryos were harvested at day E13.5, in Hanks' balanced saline solution (HBSS; GIBCO). The chicken eggs were incubated for 8 days at 37°C before the embryos (Hamburger-Hamilton stages 27–34) were removed from the eggs and rinsed in HBSS; GIBCO. Palatal shelves were dissected and cultured as previously described (Yu et al., 2008). Tgfβ3 neutralizing antibody (R&D Systems) at 10 μg/ml and PI3K inhibitor LY294002 (Calbiochem) at 1 and 10 μM final concentrations were added to the medium of cultured mouse palates, as previously described (Yu et al., 2008). Tissues were cultured for 24 h and three pairs of whole palatal shelves were processed for RNA extraction or protein analysis by western blotting. Tgfβ3 (50 ng/ml, R&D Systems) was added to the chicken palatal organ culture for 15 min to 48 h.

Madin-Darby Canine Kidney Epithelial (MDCK) cells were grown in DMEM supplemented with 10% FBS and 1% penicillin-streptomycin antibiotics. The YFP-MDCK (control) and E2A-MDCK cells were generated by transfection of the pEYFP (control) and E2A-YFP plasmids. The stable cell lines were selected by addition of 500 ug/ml gentamicin (Sigma) for 4 weeks as described before (Perez-Moreno et al., 2001).

### Snail1 siRNA transfection and treatments

The siRNA oligonucleotides specific for *Snail1* mRNA (NM_011427) were purchased from Ambion. 100 and 200 nM of siRNA in 0.1% Lipofectamine were used to transfect cells, following the manufacturer's instructions (Invitrogen). A 21-nucleotides scrambled sequence siRNA was used as a negative control. Tissues were exposed to siRNA treatment for up to 72 h and then processed for analysis. Culture medium was changed every 24 h.

### Histology and immunohistochemistry staining

Cultured palatal shelves were collected at 72 h and processed for histological analysis as previously described (Kang and Svoboda, 2002; Yu et al., 2008). The average of 20 sections' scores was calculated as the fusion score of one sample. The mean fusion score (MFS) for each treatment group was calculated. Light microscope images were captured using a Zeiss Axioplan microscope with a color RT-Spot camera.

Deparaffinized and rehydrated sections were used for immunohistochemical analysis of E-cadherin expression following standard methods. After blocking with 10% normal donkey serum/PBS, the tissues were incubated with the polyclonal antibody for E-cadherin (Cell Signaling 3195; 1:100 dilution) overnight at 4°C or 1 h at room temperature. After rinsing, the primary antibody was detected with a secondary antibody conjugated with HRP (Molecular Probes). Signal was developed with the ImmPACT DAB kit (Vector Laboratories). Nuclei were counterstained with Hematoxylin. After mounting, the images were photographed as described above.

### RNA extraction and real-time PCR

Total RNA was extracted using the RNeasy Mini Kit (Qiagen). Obtained RNA was reverse transcribed with SuperScriptII reverse-transcriptase (Invitrogen) and the resulting cDNA used for quantitative real-time PCR. The relative quantification value was calculated by the 2^−δCt^ method. All quantifications were normalized to *18s* rRNA (SuperArray) and then standardized with the negative control. Experiments were repeated at least three times. Primers user were for mouse Snail1: 5′AAACCCACTCGGATGTGAAG and 5′GAAGGAGTCCTGGCAGTGAG; for chicken: 5′CCTTTCCCGTGCAGATACAT and 3′TGCACAGGAGCACAGGATAG.

### Whole mount *in situ* hybridization

Whole-mount *in situ* hybridization analysis was performed as previously described (Ruest et al., 2004; Ruest and Clouthier, 2009). Embryos were hybridized with digoxigenin (DIG)-labeled cRNA riboprobes against *Snail1, Twist1*, and *E2A*. Stained embryos were photographed in whole-mount on an Olympus SZX16 stereoscope fitted with a digital camera.

### Immunoprecipitation

The tips of six palatal shelves pairs were dissected and lysed in buffer (50 mM Tris-HCl, pH 7.5; 150 mM NaCl; 1%Nonidet P-40) containing protease inhibitor cocktails 1 and 2 (Sigma). Total protein extracts were used. The co-immunoprecipitation was carried out using the Catch and Release reversible immunoprecipitation system following manufacturer's instructions (Millipore) with 1 μg of antibody specific for E12/47 proteins. Twist1 (sc-6269) and E12/47 (sc-763) antibodies were purchased from SantaCruz.

### Protein extraction and western blotting

Tissues or cells were lysed in the RIPA buffer (Sigma) supplemented with protease inhibitors. Protein quantification was performed using the BCA assay (Pierce). Ten microgram total protein was loaded in each well on a 4–12% NuPage Bis-Tris gel (Invitrogen). Protein was transferred onto PVDF membrane (Millipore). The membrane was incubated with polyclonal primary antibody against E-cadherin (1:1000, Cell Signaling), Snail1 (1:1000, Abcam), Twist1 (1:1000), E12/47 (1:1000) overnight at 4°C. IRDye 680 (1:5000 donkey-anti-rabbit, Licor) or IRDye 800 (1:5000, donkey-anti-goat, Licor) secondary antibodies were used to visualize the protein signals with the Odyssey infrared imaging system (Licor).

### Chromatin immunoprecipitation (ChIP) assay

The Chromatin immunoprecipitation (ChIP) assays were carried out using the EZ ChIP Chromatin Immunoprecipitation Kit following manufacturer's instructions (Upstate Biotechnology). Briefly, tips of E14.5 palatal shelves (six pairs) were fixed with formaldehyde and resuspended in lysis buffer supplemented with protease inhibitors and then processed as indicated with the E12/47 or Twist1 antibody overnight at 4°C with constant agitation. Immunoprecipitated complexes were collected and DNA released using proteinase K. Recovered DNA was used for PCR amplification with primers that were designed to cover the E-boxes present in the *Snail1* promoter region. These sites were identified using the MatInspector program from Genomatix. Primers were: E1 5′CCGTTAGGGGCTAAGTCACA and 5′AGGCCTGTTCACAACCTCAC; E2 5′GGGATGAAAGGAAGCCTAGC and 5′TCGTCCCAACGGACAAGT; E3 5′CTGGTCCTTGCTACCTCTGC and 5′TTCCAGGATGAGGTTGGTGT; E4 5′CGGTGCTTCTTCACTTCCTC and 5′ACTACCCAGGGATGCCCTAC; E5 5′TGACCGTACTGTTGGTCACG and 5′ATCATCGCACTTTCTGGCTC. Total DNA extracts were used as input controls for the PCR reactions.

### Plasmid construction

The *Snail1* expression plasmid was constructed by inserting the HindIII-BamHI fragment of the mouse *Snail1* cDNA into the pEYFP-C1 vector. The mouse *Snail1* 1.7 kb promoter flanked by the XhoI and HindIII restriction sites was cloned by PCR from genomic DNA (genome sequence NT_039201) of a CD1 mouse using high fidelity DNA polymerase (Pfu turbo, Stratagene) and confirmed by sequencing. The following primers were used:

5′ccgctcgagTGAAAAACCCTAGGTGGCAG (−1683 bp)

5′cccaagcttGCTCGCTATAGTTGGGCTTC (+64 bp).

The fragment was subcloned using the same restriction enzymes into the pGL3 luciferase vector, yielding the 1.7KSnLuc construct. pGL3 basic vector was used as a negative control. The Twist1 plasmid was described previously (Zhang et al., 2012).

### Dual luciferase assays

Subconfluent cultures of MDCK cells (2 × 10^4^ cells/well in 24-well-plate) were serum starved overnight and transfected with up to 1.2 μg of *Twist1* or *Snail1* expression plasmids along with 100 ng Snail-promoter luciferase constructs and 10 ng pRL-TK vector (Promega) as internal control. After 24-h incubation, cells were harvested and dual luciferase assay were carried out using the Dual-Glo luciferase assay system according to the manufacturer's recommendations (Promega). All the results were normalized to Renilla luciferase activities (pRL-TK). All assays were performed at least three times in triplicate. The results are mean of different experiments ± standard errors.

### Statistical analysis

Two-tailed Student's *t*-test analysis or Two-Way ANOVA were used to evaluate the statistical significance of the results. A *p* < 0.05 was considered significant. The non-parametric Kruskal-Wallis analysis of variance was used to compare MFS between groups. *P* < 0.05 was also considered statistically significant.

## Results

### Snail1 plays a role in MES degradation

**Figure 1:**
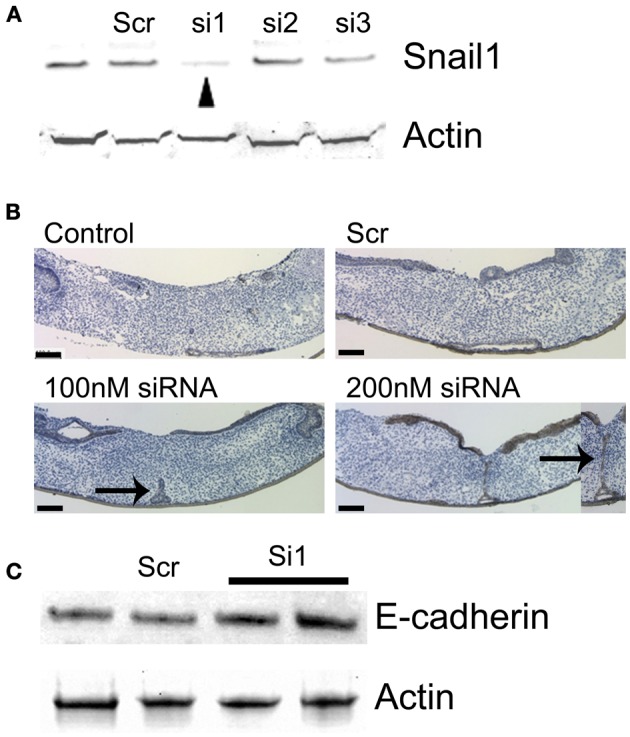
**MES degradation was blocked in presence of *Snail1* siRNA in mouse palatal organ culture. (A)** Snail1 siRNA 1 (si1) decreased Snail1 protein levels in mouse palate as revealed by western blotting (arrowhead). Actin was used a loading control. The other screened siRNAs (si2 and si3) were not efficient. Scrambled sequence siRNA (scr) was used as control. **(B)** *Snail1* siRNA decreased mouse palatal fusion in a dose-dependent manner. Palatal shelves were cultured for 72 h with or without *Snail1* siRNA. Sections were stained with E-cadherin antibody (brown) to detect epithelial cells. The tissues were counterstained with hematoxylin (blue). In control and scr control groups, the palates completely fused and no E-cadherin stained cells were found in the midline. In presence of 100 and 200 nM *Snail1* siRNA, E-cadherin stained cells were in the epithelial triangle (100 nM siRNA, arrow) and midline seam (arrow in 200 nM siRNA inset). Inset: higher magnification of the midline seam (200 nM siRNA). Scale bars = 100 μm. **(C)** Western blotting analysis showing that E-cadherin protein levels are elevated in MDCK cells transfected with the *Snail1* siRNA.

In order to examine the function of Snail1 during MES degradation, we specifically down regulated *Snail1* expression by using siRNA in palatal organ culture. Downregulation efficiency of several *Snail1* siRNAs were tested by Western blot (Figure 1A). siRNA1 (si1) suppressed Snail1 protein expression efficiently and was used in all of the following palatal organ culture experiments. A scrambled sequence siRNA (scr) was used as a negative control. The palatal shelves were maintained with or without *Snail1* siRNA for 72 h before processing for histological evaluation of palatal fusion (Figure 1B). The immunohistochemical staining of E-cadherin was used to detect epithelial cells in the midline. In control palates and scrambled siRNA groups the palate completely fused without evidence of MES E-cadherin expressing epithelial cells in the mesenchyme (Figure 1B). In palatal shelves treated with 100 nM *Snail1* siRNA, the epithelial seam broke down and degraded but triangular clusters of E-cadherin-positive epithelial cells were found primarily on the nasal side (Figure 1B, arrow). In palatal shelves treated with 200 nM *Snail1* siRNA, the E-cadherin positive epithelial seam remained mostly intact (Figure 1B, inset 200 nM siRNA). The palatal shelf size between the four treatment groups was similar (Figure 1B, scale bars).

The degree of palatal fusion was quantified with a scaling system, termed the MFS, as previously described (Yu et al., 2008). Briefly, a score of 5 equals complete palatal fusion. Lower MFS indicate more epithelial cells in the midline seam, indicating that fusion was blocked or decreased (Table 1). In control and scrambled siRNA groups, most sections scored 4–5, indicating complete fusion of the palatal shelves. In presence of *Snail1* siRNA at both 100 and 200 nM, the MFS decreased to 3.4, suggesting epithelial islands or seam remnants were found. The non-paprametric Kruskal-Wallis analysis of variance was used to compare MFS between groups. The MFS in 100 and 200 nM *Snail1* siRNA group were significantly different from the control and scrambled siRNA control groups, indicating that the degradation of MES was reduced or delayed in presence of *Snail1* siRNA *in vitro*.

**Table 1:** **Mean fusion score of Snail1 siRNA-treated cultured mouse palates**.

	**Non-fusion**	**Partial fusion**		**Complete fusion**	***n***	**MFS**
	**1–2**	**2–3**	**3–4**	**4–5**		
Control	0	0	0	6	6	4.6
Scrambled siRNA	0	0	0	7	7	4.3
Snail1 siRNA 100 nM	0	2	2	1	5	3.4^*^
Snail1 siRNA 200 nM	0	1	4	2	7	3.4^*^

n ≈ 20 sections/sample;

* p < 0.05 (Kruskal–Wallis).

Snail1 is a known repressor of *E-cadherin* expression (Batlle et al., 2000; Cano et al., 2000; Oram and Gridley, 2005). We performed the cognate experiment to confirm (1) the role of Snail1 on E-cadherin abundance and (2) the effect observed in MEE occurs in other epithelial cells. We examined whether reducing Snail1 abundance in cultured MDCK epithelial cells altered E-cadherin protein levels (Figure 1C). In the cells transfected with the *Snail1* siRNA, E-cadherin levels increased about two-folds, confirming that Snail1 regulates *E-cadherin* levels in epithelial cells (Figure 1C).

### *Snail1* mRNA expression is Tgfβ3 and PI-3 kinase (PI3K) signaling-dependent

**Figure 2:**
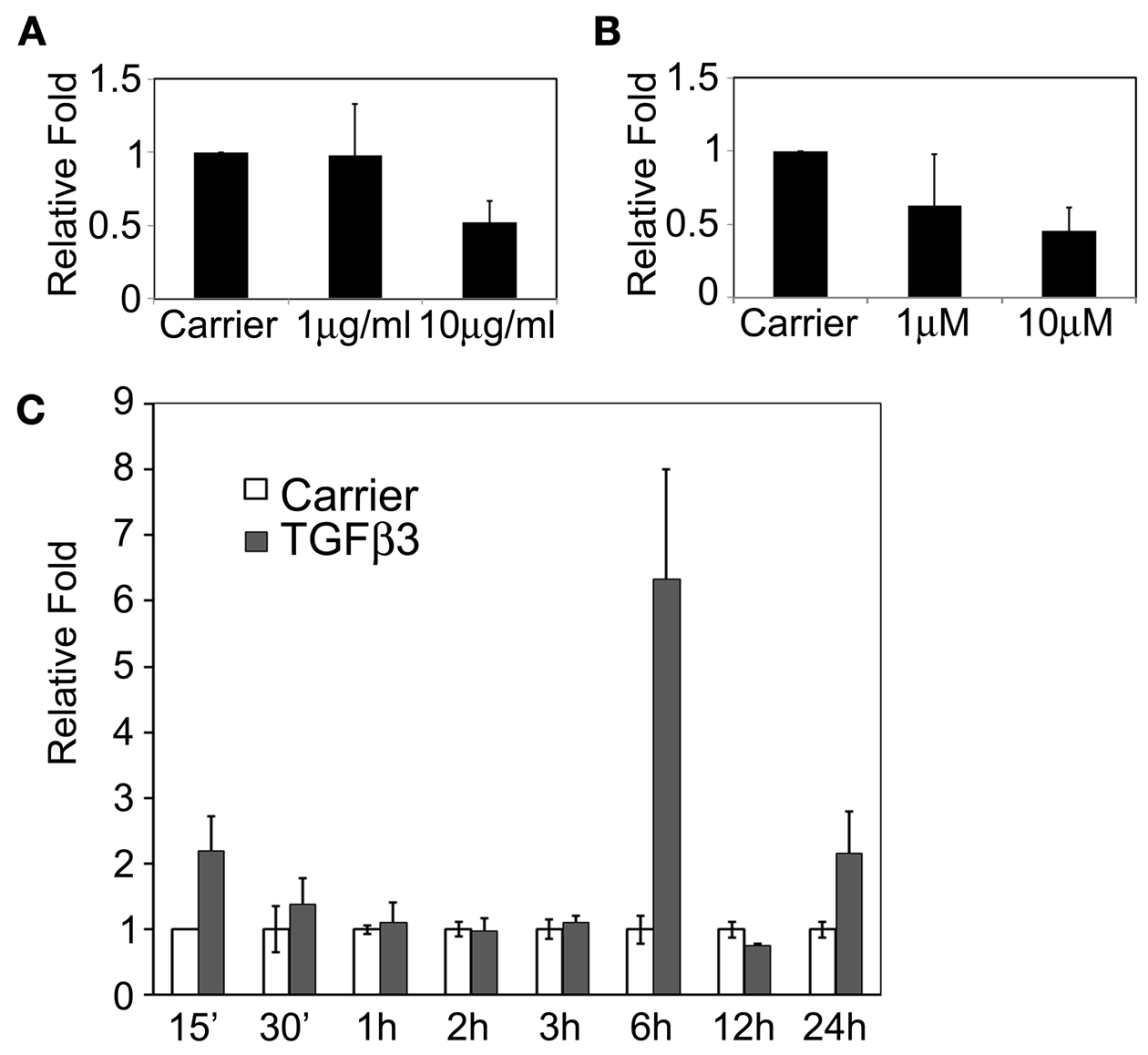
**Snail1 expression responses to Tgfβ3 and PI3K signaling in cultured mouse and chicken palatal shelves. (A)** Quantitative RT-PCR of *Snail1* expression in cultured murine shelves following the blockage of Tgfβ3 signaling with neutralizing antibodies for 24 h (10 μg/ml, *p* = 0.0175). **(B)** Quantitative RT-PCR of *Snail1* expression in mouse palate following treatment with the PI3K inhibitor LY294001 for 24 h (10 μM, *p* = 0.0396). Data represented mean ± SE; *n* = 3. **(C)** RT-PCR Analysis of Snail1 expression in cultured chicken palatal shelves following treatment with Tgfβ3. Control samples were treated with the carrier and normalized to one. Data represented mean ± SEM; *n* = 3 (*p* = 0.0008 at 6 h).

It has been established that *Tgf*β*3* and *PI3K* are required for murine palatal fusion (Kaartinen et al., 1995; Proetzel et al., 1995; Kang and Svoboda, 2002). Tgfβ3 signaling is likely mediating PI3K activation in MEE cells. To explore if these connected signaling pathways regulate *Snail1* expression during palatal fusion, we used a Tgfβ3 neutralizing antibody and PI3K inhibitor in the palatal organ culture system. In presence of 1 μg/ml Tgfβ3 neutralizing antibody, *Snail1* expression did not change (Figure 2A) but in presence of 10 μg/ml Tgfβ3 neutralizing antibody, *Snail1* expression was significantly decreased (*p* = 0.0175). In palates treated with the PI3K inhibitor LY294002, *Snail1* expression decreased in a dose-dependent manner (Figure 2B). However, only the higher dose, 10 μM of PI3K inhibitor, produced a significant decrease in *Snail1* expression (*p* = 0.0396). These data were indirectly suggesting that Tgfβ3 was regulating the expression of the gene. To test whether Tgfβ3 was directly regulating *Snail1* expression, we used chicken palates. These palates do not normally fuse since Tgfβ3 is not expressed in the MEE, but they fuse when treated in culture with the growth factor (Sun et al., 1998). We used this model to determine if *Snail1* expression changed in response to exogenous Tgfβ3. Six hours after Tgfβ3 treatment, the *Snail1* expression transiently increased approximately six-folds (*p* = 0.0008) (Figure 2C). The response appeared temporally limited but the results were indicating that Tgfβ3 can upregulate *Snail1* expression.

### Twist1 regulates *Snail1* promoter activity

Although *Twist1, Snail1*, and *E2A* genes trigger EMT in different biological contexts independently, evidence supports a differential and hierarchical role for these repressors during the transformation process (Peinado et al., 2004). They may form a complex signaling network to regulate the transition process (Peinado et al., 2007). *Tgfβ* factors promote the expression of *Snai11, Snail2, Zeb1, Zeb2*, and *Twist1* in cell- or tissue-dependent contexts (Zavadil and Bottinger, 2005; Thuault et al., 2006).

**Figure 3:**
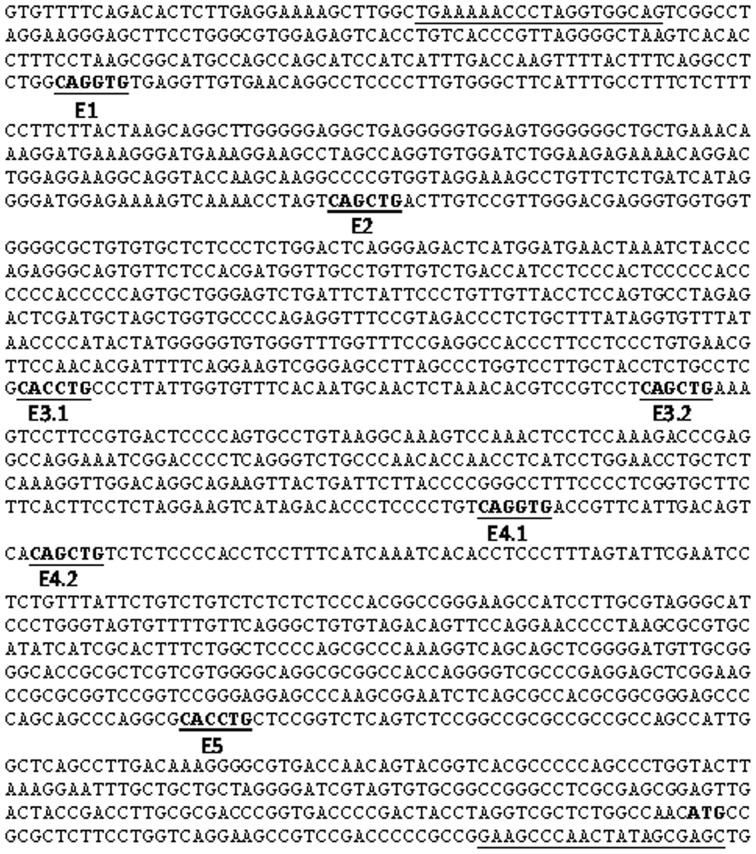
**Mouse *Snail1* promoter sequence.** The mouse Snail1 promoter was cloned using the oligo primers underlined in the sequence. The ATG transcription start site, located toward the end of the sequence, is bolded. E-boxes are bolded and underlined and numbered in a 5′ to 3′ direction.

The transcription factor Twist1 plays both positive and negative roles in regulation of embryonic morphogenesis and cell differentiation (O'Rourke and Tam, 2002). Twist1 can form functional homodimers as well as heterodimers with ubiquitously expressed bHLH E protein, such as *E2A* gene products E12 and E47. In a previous study, we demonstrated that Tgfβ3 regulates the expression of *Twist1*. Since *Snail1* activation by Tgfβ3 was delayed, we investigated whether Twist1 was needed for *Snail1* expression. When exploring the *Snail1* promoter region, we detected the presence of 7 CANNTG E-boxes upstream of the transcription start site (Figure 3). In a 5′ to 3′ direction, these E-boxes are named E1 (−1550/−1545), E2 (−1289/−1284), E3.1 (−893/−888), E3.2 (−843/−838), E4.1 (−617/−612), E4.2 (−593/−588) and E5 (−120/−115). These E-boxes are presumably E47 and Twist1 binding sites, suggesting that these factors may directly bind the promoter and regulate *Snail1* transcription. Thus, we hypothesized that Twist1 cooperates with E47 and acts upstream of *Snail1* during palatal EMT.

**Figure 4:**
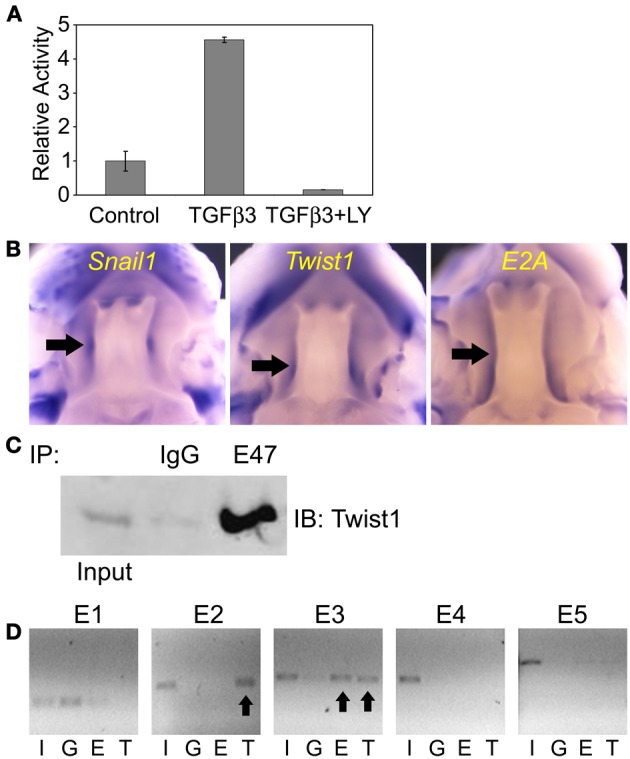
**Twist and E2A dimerize and bind to the mouse *Snail1* promoter in the developing palate. (A)** Luciferase assay results in MDCK cells with the *Snail1* promoter showing that after 24 h, Tgfβ3 signaling stimulated the Snail1 promoter (*p* = 0.0413) but the activation is inhibited by the PI3K inhibitor LY294002 (*p* = 0.0030). (**B)** Analysis of *Snail1, E2A*, and *Twist1* expression in palatal shelves (black arrows) in E13.5 mouse embryos using whole mount *in situ* hybridization. **(C)** Co-immunoprecipitation results showing that Twist1 and E47 dimerize in pre-fused E14.0 mouse palatal shelves *in vivo*. Immunoprecipitation (IP) with the E47 antibody and immunoblotting (IB) with the Twist1 antibody demonstrated that the two proteins interact prior to palatal fusion. Unspecific rabbit IgG was used as a negative IP control. **(D)** ChIP results revealing that Twist1 and E47 bind selective E-boxes of the *Snail1* promoter (black arrows) from mouse palatal shelves *in vivo*. I, input; G, IgG control; E, E47 antibody; T, Twist1 antibody.

We first tested whether Tgfβ3 regulated the *Snail1* promoter activity. We cloned the mouse *Snail1* promoter into a luciferase reporter vector. The 1.7 Kb promoter-luciferase constructs named *1.7KSnLuc* was transfected in the epithelial MDCK cells. These cells were selected based on their similar behavior to palate epithelial cells as described above. The 1.7 Kb promoter showed a significant activation upon Tgfβ3 stimulation (*p* = 0.0413) (Figure 4A). That response was blocked when the PI3K inhibitor LY294002 was added to the medium (*p* = 0.030). We then examined the expression of the *Snail1, Twist1*, and *E2A* genes in the E13.5 mouse palate by whole mount *in situ* hybridization. The expression of these three genes overlapped in the palatal shelves (Figure 4B). All three genes were expressed in the palatal shelf along its entire length. A gradient of expression was also observed for *Twist1*, with lower expression in anterior shelves and higher at the posterior area. E2A expression was lower in the lip/nose pad area, suggesting that functions observed in the developing palate may differ in the developing lip. In addition, we used co-immunoprecipitation to test whether Twist1 and E47 interact in the palatal shelves. We used the tip of the touching E14.0 palatal shelves for protein extractions. E47 antibody was incubated with the protein lysate and Twist1 western blotting was used to detect if the proteins co-immunoprecipitated. Twist1 protein was detected when E47 was immunoprecipitated (Figure 4C). Our results suggested that Twist1 physically interacts with E47 in the palate tissue prior to fusion.

In order to test whether E2A (E12/E47) and Twist1 proteins can bind to the *Snai11* promoter, we used the ChIP assay on the pre-fusion palatal shelves. We dissected the tip of the mouse palatal shelves where both transcription factors are expressed prior to fusion. Five pairs of primers targeting the different E-boxes on the *Snail1* promoter, with no distinction between E3.1 and E3.2 or E4.1 and E4.2 since each pair's E-boxes were close. After immunoprecipitation of the DNA-protein complexes with the Twist1 and Snail1 antibodies, PCR was used to amplify the presumptive targeted E-box regions. Our results show that E47 and Twist1 can both bind to the E3 region (Figure 4D). Only Twist1 bound the E2 region. Twist1 or E47 did not interact with the other E-boxes in the mouse palatal shelves.

**Figure 5:**
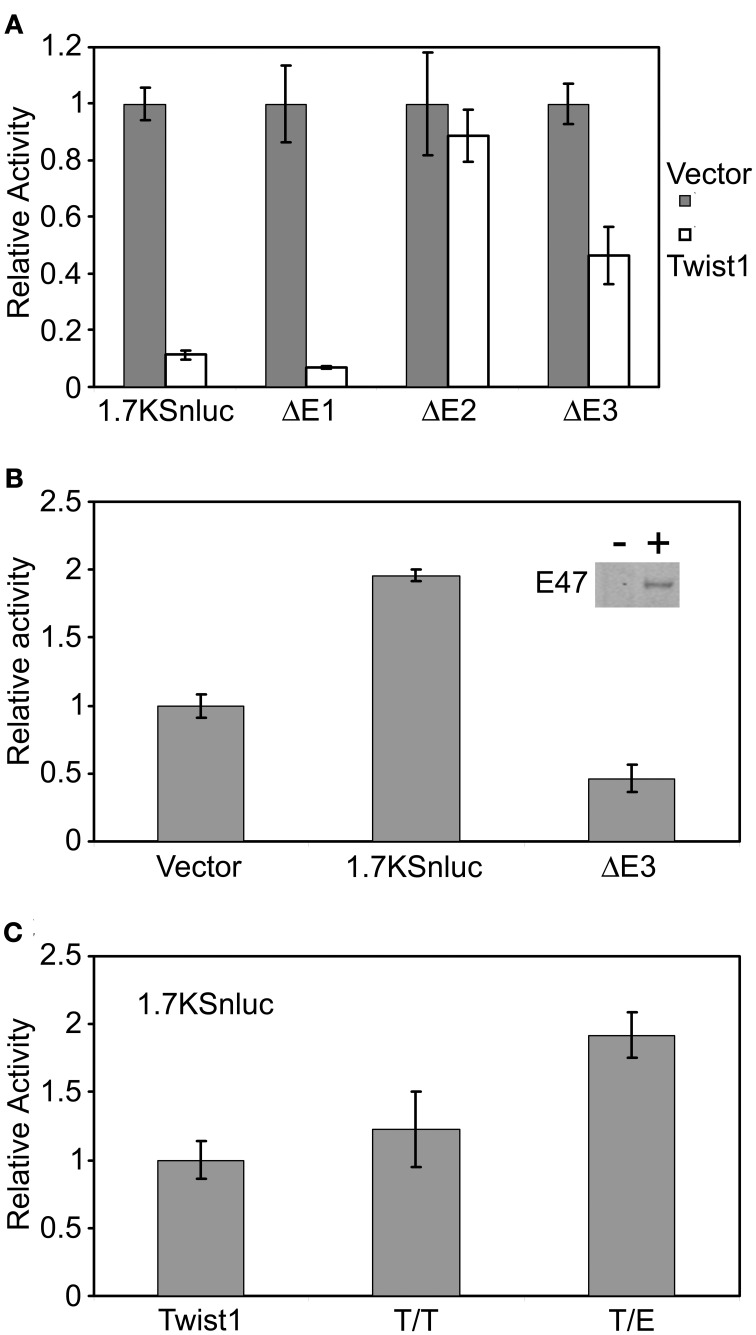
**Twist1 regulates the mouse *Snail1* promoter activity by dimerizing with E-proteins. (A)** Luciferase assay using the *Snail1* promoter results revealed that Twist1 alone cannot activate the *Snail1* promoter but rather represses the activity of the promoter (*p* = 5.73E-05). This repression was released when the E2 site was removed (ΔE2). **(B)** Twist1 increased *Snail1* promoter activity in MDCK-E2A cells, indicating that the interaction between Twist1 and E47 was needed to activate the *Snail1* promoter. Removal of the E3 region (ΔE3) abolished the activation (*p* = 0.0048). Inset: western blot analysis of E47 expression in stably transfected cells (10 μg of protein loaded). **(C)** Luciferase assay results confirming that the interaction between Twist1 and E47 was necessary to activate the *Snail1* promoter (*p* = 0.00096). Forced Twist1/Twist1 (T/T) homodimers and Twist1/E47 (T/E) heterodimers encoding vectors were transfected in the cultured MDCK cells and luciferase assay results compared to the control Twist1 expression vector. Luciferase activity was measured 24 h after transfection. Data represented mean ± SEM; *n* = 3. An empty expression vector was used as a control for these experiments.

However, the ChIP results could not distinguish from Twist1 or E protein response in the epithelial, mesenchymal cells or subpopulations of epithelial cells. Based on our results and those from Yu et al. (2008), the response in the MEE cells is likely triggered by Tgfβ3 inducing *Twist1* expression in these cells. To identify how Twist1 regulates the *Snail1* promoter activity in epithelial cells, we used the cloned mouse *Snail1* promoter into the luciferase reporter vector. The promoter-luciferase constructs *1.7KSnLuc* transfected in MDCK cells was significantly repressed in presence of Twist1 (*p* = 5.73E-05) (Figure 5A). These results are in agreement with others suggesting that often Twist1 acts as a transcriptional repressor (Spicer et al., 1996; Yin et al., 1997). When E1 was removed, the same repression was observed (*p* = 0.0003). When the E2 site was removed, the repression was abolished but activation was not observed. Only Twist1 bound the E2 site in the ChIP assays. When the E3 region bound by both E47 and Twist1 was removed, a significant decrease in luciferase activity was noted (*p* = 0.0198). These results indicated the possible inhibitory effects on *Snail1* expression exerted by Twist1. Removing the E2 site released the repression, but removing the E3 region possibly blocked the activation of the luciferase expression. To test whether Twist1 and E2A proteins synergistically regulate *Snail1* promoter activity, the luciferase vector was transfected along with *Twist1* in MDCK cells stably expressing E47 protein. These stably transfected cells allowed testing the different conditions while maintaining a steady level of E47 protein. In these cells, a Twist1 response was observed (*p* = 0.0048) (Figure 5B). These data suggested that Twist1 and E47 were co-regulating *Snail1* expression. The ChIP results indicated that the E3 region was bound by both Twist1 and E47. When the E3 region was removed, the luciferase response significantly decreased (*p* = 0.02) (Figure 5B), suggesting that the site was essential to regulate *Snail1* expression by Twist1 and E proteins.

Since Twist1 can form either Twist1/Twist1 (T/T) homodimer or Twist1/E-protein (T/E) heterodimer in different biological events, our results suggested that the regulation of Twist1 on *Snail1* promoter activity depends on the ratio of T/T to T/E dimers. To test this hypothesis, we transfected the MDCK cells with plasmids which expressed “forced dimers” of either T/T or T/E. These constructs have been described previously (Connerney et al., 2006). The 1.7KSnLuc response increased two-fold in response to T/E dimers (*p* = 0.00096) whereas no obvious change was observed in response to the T/T dimer in comparison with cells transfected with *Twist1* alone (Figure 5C). These results indicated that Twist1 dimerizes with E-proteins to activate the *Snail1* promoter activity.

## Discussion

### Twist1 signals through Snail1 to downregulate *E-cadherin* during MEE cell migration

The function of Snail genes is best known for their direct repression of *E-cadherin* expression (Cano et al., 2000; Nieto, 2002). Snail genes have additional cellular functions, such as cell survival, cell adhesion, and migration (Barrallo-Gimeno and Nieto, 2005). Ectopic expression of *Snail1* in the MDCK epithelial cell line promotes resistance to apoptosis (Escriva et al., 2008). Previously, *Snail1* was localized to a small subgroup of palatal MEE cells (Martinez-Alvarez et al., 2004). However, its role in palatal fusion is not clear. In this study the expression of *Snail1* was down regulated with a specific siRNA in palatal organ cultures. In treated cultured shelves, the palates failed to completely fuse and epithelial remnants were present after the 72 h culture period. We used E-cadherin as a marker to establish that the cells retained an epithelial phenotype when *Snail1* function was suppressed. Our results suggested that *Snail1* was at least required for E-cadherin suppression during MES degradation, in line with other studies (Cano et al., 2000; Medici et al., 2008). Since conditional *Snail1* mutant mouse embryos do not develop a cleft palate, our culture results with the siRNA indicate that Snail1 is needed for palatal fusion but fusion is delayed when absent due to compensatory effects by Snail2 (Murray et al., 2007).

In our study, we found increased *Snail1* expression in response to Tgfβ3 stimulation and decreased expression when Tgfβ3 signaling was reduced. Our previous study indicated that Tgfβ3 through PI3K activation regulates *Twist1* expression (Yu et al., 2008). In this study we show that Twist1 regulates *Snail1* expression. The delay observed in the Tgfβ3 response in chicken palate may represent the time needed to activate *Twist1* expression. Our results suggest that *Snail1* activation by Tgfβ3 may be sequential to *Twist1* activation. Reduction of *Snail1* expression to blocked *Tgf*β*3* or PI3K signaling was not as great as expected. This may be due to the fine-tuning feedback mechanism of *Snail1* regulation with Snail1 binding its own promoter region to create a negative loop controlling its own expression (Peiro et al., 2006). In addition, signaling pathways other than Tgfβ3 also contribute to *Snail1* regulation (Barrallo-Gimeno and Nieto, 2005), many of which play important roles in palatal fusion as well. The activity of the *Snail1* promoter during EMT is dependent on *Erk2* and Gsk-3β/NFkB pathway activity (Barbera et al., 2004). PI3K activity also contributes to *Snail1* transcription and promoter activity (Peinado et al., 2003), possibly acting in the same signal pathway as GSK3β/NFkb. In our study, *Snail1* mRNA levels responded to both Tgfβ3 and PI3K, suggesting that Tgfβ3 may signal through the PI3K/Gsk3β route to regulate *Snail1* expression levels during MEE transdifferentiation, migration or death.

### Twist1 regulates *Snail1* promoter activity in collaboration with E-proteins

**Figure 6:**
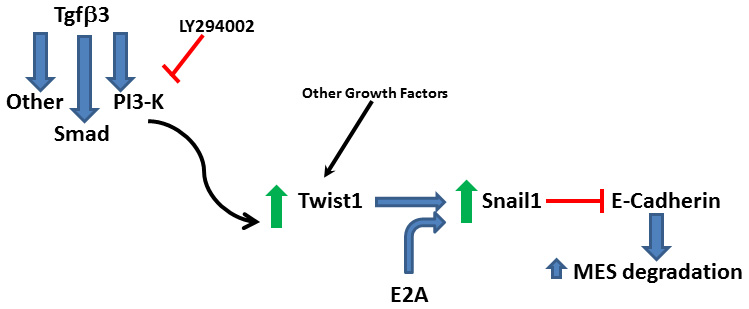
**Proposed signaling pathway.** Twist1 is upregulated by Tgfβ3 through signaling molecules including PI3-kinase and it dimerizes with E2A proteins to upregulate Snail1 to repress E-cadherin to promote the degradation of the medial edge seam (MES).

We demonstrated that Twist1 binds the *Snail1* promoter and regulates its activity by recruiting E-proteins (E12/E47), which are also expressed during palatal fusion. Without the synergy with the E-proteins, Twist1 represses Snail1 expression, probably indicating a spatial or temporal regulatory mechanism (Figure 6). Our *in situ* results show that *Twist1* expression is higher in the posterior half of the developing palate, where fusion occurs later. The *E2A* gene is more uniformly expressed with a slightly higher expression at the posterior ends of the shelves. The different levels of expression may have an impact on the dimerization of Twist1 and E-proteins and cellular expression and *in vivo* protein-protein interaction analyses could eventually help resolve this issue. However, based on the respective gene expression patterns, we hypothesize that the higher *Twist1* expression in the posterior half of the palate would favor the formation of Twist1/Twist1 dimers instead of Twist1/E-protein dimmers that would be favored more anteriorly. The skewed ratios would favor the repression of *Snail1* expression in the posterior palate and promote its expression more anteriorly. Our *in situ* analysis of *Snail1* expression supports this hypothesis since the expression of the gene is higher where *Twist1* expression is intermediate. In the anterior palate where Twist1 expression is lower, Snail1 expression is also reduced but not absent, suggesting the presence of other unidentified regulatory mechanisms. The results indicate that the balance between Twist1 and E-proteins regulates the spatial, temporal and expression levels of *Snail1* and can explain why both E2 and E3 sites were precipitated from whole palatal shelf protein extracts.

Twist1 and Snail proteins are involved in EMT. We think that the regulation of *Snail1* expression by Twist1 is tightly regulated based on the short Twist1 protein presence in the MEE (Yu et al., 2008; Kitase et al., 2011). Targeting these factors is likely affecting the EMT associated with palatal fusion and the consequent apoptotic death of the transformed cells (Jin and Ding, 2006; Ahmed et al., 2007), as evidenced by the remaining MES cells in our treated samples. Apoptosis is important for palatal fusion and appears to be regulated by Tgfβ3 signaling (Martinez-Alvarez et al., 2000). In the *Tgf*β*3* mutant embryos, compensation by Tgfβ1 promotes the expression of *Snail1* and *2* in the MEE (Martinez-Alvarez et al., 2004). Elevated Snail gene expression correlates with the resistance to apoptosis in the MEE cells and affects EMT. However, in the *Snail1^+/−^; Snail2^−/−^* mouse embryos, apoptosis resistance was observed (Murray et al., 2007). Our results showing that MES cells remain in the midline are in agreement with these last results indicating that Snail1 function may be needed for apoptosis. It appears that EMT precedes apoptosis (Jin and Ding, 2006; Ahmed et al., 2007), at least in some of the MES cells. This process is also regulated by Tgfβ3 (Martinez-Alvarez et al., 2004). When this growth factor is absent, MEE cells fail to generate lamellipodia and filopodia (Taya et al., 1999), characteristic structures of migratory mesenchymal cells. In palatal fusion, Twist1 may cooperate with E-proteins to activate *Snail1* expression and regulate the *E-cadherin* expression (Figure 6), while using other factors to regulate the cell migratory behavior. Thus, future experiments should explore the theory that Twist1 can modify the filopodia-like structures on the apical surface of the MEE cells through a small GTPase, such as Cdc42.

Several families of transcription factors other than Snail1, such as the ZEB family, independently induce EMT in different contexts (Yang et al., 2004; Liu et al., 2008; Medici et al., 2008). However, the complex and multifaceted process that defines EMT result from a plexus of changes in transcriptional regulation (Peinado et al., 2007). It is very plausible that a number of such EMT-promoting factors may act together as an EMT signaling network (Yang et al., 2006). Snail1 has been implicated in the initial migratory phenotype and considered as an early marker of EMT that sometimes contributes to the induction of other factors. By contrast, Snail2, Zeb1, Zeb2, and/or Twist1 could be responsible for the maintenance of migratory cell behavior (Peinado et al., 2007). During neural crest development in vertebrates, expression of *Snail1* and *Snail2* occurs at the neural plate border where *Twist1* is also expressed, and all three transcription factors play critical roles in neural crest formation (Meulemans and Bronner-Fraser, 2004). However, in Drosophila, *Twist* induces the expression of the transcription factor *Snail* to allow invagination and mesoderm differentiation (Furlong et al., 2001). In *Twist* or *Snail* mutant Drosophila embryos, the ventral invagination is largely abolished. The double mutant has the strongest phenotype, suggesting that the two genes have both overlapping and distinct functions (Leptin, 1999). A key function of Twist is to collaborate with Dorsal to optimally activate the expression of *Snail* (Ip and Gridley, 2002). Moreover, genetic rescue experiments demonstrated that forced expression of *Snail* in the absence of *Twist*, but not vice versa, can promote ventral-cell invagination. Therefore, it is suggested that although *Twist* and *Snail* may each have non-overlapping functions, *Snail* has a more direct role in regulating downstream events leading to gastrulation (Ip and Gridley, 2002). It is unclear whether similar mechanisms are also involved with Twist1 and Snail1 during palate development.

In summary, we established that Snail1 expression is needed to repress *E-cadherin* expression to facilitate the MES degradation. Furthermore, we revealed that Tgfβ3 response requires the *Snail1* activation by Twist1, establishing a sequential mechanism leading to MES degradation (Figure 6). We anticipate that this degradation possibly involves the transdifferentiation of palatal epithelial cells into mesenchymal cells before their migration away from the seam or death because of the known functions of Twist1 and Snail1 in EMT.

### Conflict of interest statement

The authors declare that the research was conducted in the absence of any commercial or financial relationships that could be construed as a potential conflict of interest.
